# The 2016 WHO classification and diagnostic criteria for myeloproliferative neoplasms: document summary and in-depth discussion

**DOI:** 10.1038/s41408-018-0054-y

**Published:** 2018-02-09

**Authors:** Tiziano Barbui, Jürgen Thiele, Heinz Gisslinger, Hans Michael Kvasnicka, Alessandro M. Vannucchi, Paola Guglielmelli, Attilio Orazi, Ayalew Tefferi

**Affiliations:** 1 FROM Research Foundation, Papa Giovanni XXIII Hospital, Bergamo, Italy; 2 Institute of Pathology, University of Cologne, Cologne, Germany; 3 Medical University of Vienna, Vienna, Austria; 4 Senckenberg Institute of Pathology, University of Frankfurt, Frankfurt, Germany; 5 University of Florence, Florence, Italy; 6 CRIMM-Centro Ricerca e Innovazione delle Malattie Mieloproliferative, Azienda Ospedaliera-Universitaria Careggi, Department of Experimental and Clinical Medicine, University of Florence, Florence, Italy; 7 Department of Pathology and Laboratory Medicine, Weill Cornell Medical College, New York, NY USA; 8 Mayo Clinic, Rochester, MN USA

## Abstract

The new edition of the 2016 World Health Organization (WHO) classification system for tumors of the hematopoietic and lymphoid tissues was published in September 2017. Under the category of myeloproliferative neoplasms (MPNs), the revised document includes seven subcategories: chronic myeloid leukemia, chronic neutrophilic leukemia, polycythemia vera (PV), primary myelofibrosis (PMF), essential thrombocythemia (ET), chronic eosinophilic leukemia-not otherwise specified and MPN, unclassifiable (MPN-U); of note, mastocytosis is no longer classified under the MPN category. In the current review, we focus on the diagnostic criteria for *JAK2*/*CALR*/*MPL* mutation-related MPNs: PV, ET, and PMF. In this regard, the 2016 changes were aimed at facilitating the distinction between masked PV and *JAK2*-mutated ET and between prefibrotic/early and overtly fibrotic PMF. In the current communication, we (i) provide practically useful resource tables and graphs on the new diagnostic criteria including outcome, (ii) elaborate on the rationale for the 2016 changes, (iii) discuss the complementary role of mutation screening, (iv) address ongoing controversies and propose solutions, (v) attend to the challenges of applying WHO criteria in routine clinical practice, and (vi) outline future directions from the perspectives of the clinical pathologist.

## Introduction

The 2016 revised “Blue Book”, the official document of the World Health Organization (WHO) classification system for tumors of the hematopoietic and lymphoid tissues, has now been published^1^. The current communication focuses on myeloproliferative neoplasms (MPNs) and provides a more comprehensive syllabus that is organized into eight sections: section one starts with a list and brief overview of the seven clinic-pathologic entities that currently comprise the WHO MPN category; section two provides practically useful resource tables and graphs on the 2016 WHO diagnostic criteria and outcome for the *JAK2*/*CALR*/*MPL* mutation-related MPNs, including polycythemia vera (PV), essential thrombocythemia (ET), and primary myelofibrosis (PMF), including particularly prefibrotic/early PMF (pre-PMF); section three addresses the rationale behind the 2016 changes in the diagnostic criteria for PV, ET, PMF; section four attends to the complementary role of mutation screening and its limitations for diagnostic purposes; section five highlights current controversies regarding the new diagnostic criteria, especially in regards to diagnosis of PV and pre-PMF; section six offers proposed solutions for currently ongoing controversies; section seven considers the challenges in applying the WHO criteria in routine clinical practice, and discusses future directions from the perspective of the physician scientist; section eight outlines solutions and future directions from the perspective of the clinical pathologist.

### The 2016 WHO sub-categorization of MPNs and brief overview of the diagnostic criteria for CML, CNL, CEL-NOS, and MPN-U

Morphology remains the central distinguishing feature in the 2016 WHO system for classification of tumors of the hematopoietic and lymphoid tissues, although mutation screening is increasingly being utilized for confirmation of morphologic diagnosis and, at times, for directing the diagnostic process^1, 2^.

Myeloid neoplasms continue to be organized into acute myeloid leukemia and chronic myeloid neoplasms, based primarily on the percentage of peripheral blood or bone marrow (BM) blasts. Chronic myeloid neoplasms are in turn classified into four operational categories: myelodysplastic syndromes (MDS), MPNs, MDS/MPN overlap and myeloid/lymphoid neoplasms with eosinophilia and recurrent rearrangements of *PDGFRA*, *PDGFRB*, and *FGFR1* or *PMC1-JAK2*; the latter mutations correspond to 5q33, 4q12, 8p11.2 or t(8;9)(p22;p24.1) cytogenetic abnormalities, respectively. MPNs are generally distinguished from both MDS and MDS/MPN, by the absence of morphologic dysplasia, which includes dyserythropoiesis and dysgranulopoiesis and monocytosis.

The 2016 WHO category of MPNs includes the three major subcategories of *JAK2*/*CALR*/*MPL* mutation-related MPNs (i.e., PV, ET, and PMF), as well as four other clinicopathologic entities: chronic myeloid leukemia (CML), chronic neutrophilic leukemia (CNL), chronic eosinophilic leukemia, not otherwise specified (CEL-NOS) and MPN, unclassifiable (MPN-U). The *JAK2*/*CALR*/*MPL* mutation-related MPNs constitute the main focus of discussion in the current review and are further elaborated in sections 2 through 8^1, 2^.

The diagnostic hallmark of CML is the invariable presence of the *BCR-ABL1* mutation. However, minor *BCR-ABL1*-harboring sub-clones are sometimes detected in other myeloid neoplasms, including the *JAK2*/*CALR*/*MPL*-mutated MPNs, and do not necessarily alter the morphologically prominent diagnosis^3^. Similarly, JAK2-mutated clones are sometimes detected in patients with CML, especially after successful treatment with imatinib^4^.

CNL constitutes clonal proliferation of mature neutrophils and is usually associated with activating mutations (mostly T618I) of the gene (*CSF3R*) encoding for the receptor for granulocyte colony-stimulating factor, also known as colony-stimulating factor 3^5^. *CSF3R* mutations appear to be specific to WHO-defined CNL^6^. Diagnosis of CNL requires exclusion other causes of neutrophilia, including infections and inflammatory processes, metastatic cancer, and plasma cell neoplasms with secondary neutrophilia^7^. Mature-appearing neutrophilia also occurs in other myeloid malignancies, including atypical CML, *BCR-ABL1*-negative (aCML) and chronic myelomonocytic leukemia. Accordingly, the 2016 WHO diagnostic criteria for CNL are designed to exclude the possibilities of both secondary and clonal neutrophilia associated with myeloid malignancies other than CNL: leukocytosis (≥25 × 10^9^/L), ≥80% segmented/band neutrophils, <10% immature myeloid cells, <1% circulating blasts and absence of dysgranulopoiesis or monocytosis (monocyte count <1 × 10^9^/L). In clinical practice, the presence of a membrane proximal *CSF3R* mutation in a patient with neutrophilic granulocytosis should be sufficient for the diagnosis of CNL, regardless of the degree of leukocytosis.

CEL-NOS constitutes clonal eosinophilia and is considered in the presence of ≥1.5 × 10^9^/L absolute eosinophil count in the peripheral blood that is accompanied by either the presence of myeloblast excess (either >2% in the peripheral blood or 5–19% in the bone marrow) or presence of a clonal cytogenetic abnormality^8^. Cytogenetic abnormalities in CEL-NOS include trisomy 8 (the most frequent), t(10;11)(p14;q21), and t(7;12)(q11;p11). Targeted next-generation sequencing studies have recently suggested the possibility of re-classifying some cases of “hypereosinophilic syndrome” as CEL-NOS^9, 10^. Unlike the case with *PDGFRA/B*-rearranged myeloid/lymphoid neoplasms with eosinophilia, imatinib therapy is ineffective in CEL-NOS.

The WHO MPN sub-category of MPN-U includes MPN-like neoplasms that cannot be clearly classified as one of the other six subcategories of MPNs. Patients with MPN-U might present with otherwise unexplained thrombosis, especially splanchnic vein thrombosis^11^, which is associated with normal blood count.

### Practically useful resource tables and graphs on the 2016 WHO diagnostic criteria for PV, ET, and PMF including particularly pre-PMF

**Fig. 1:**
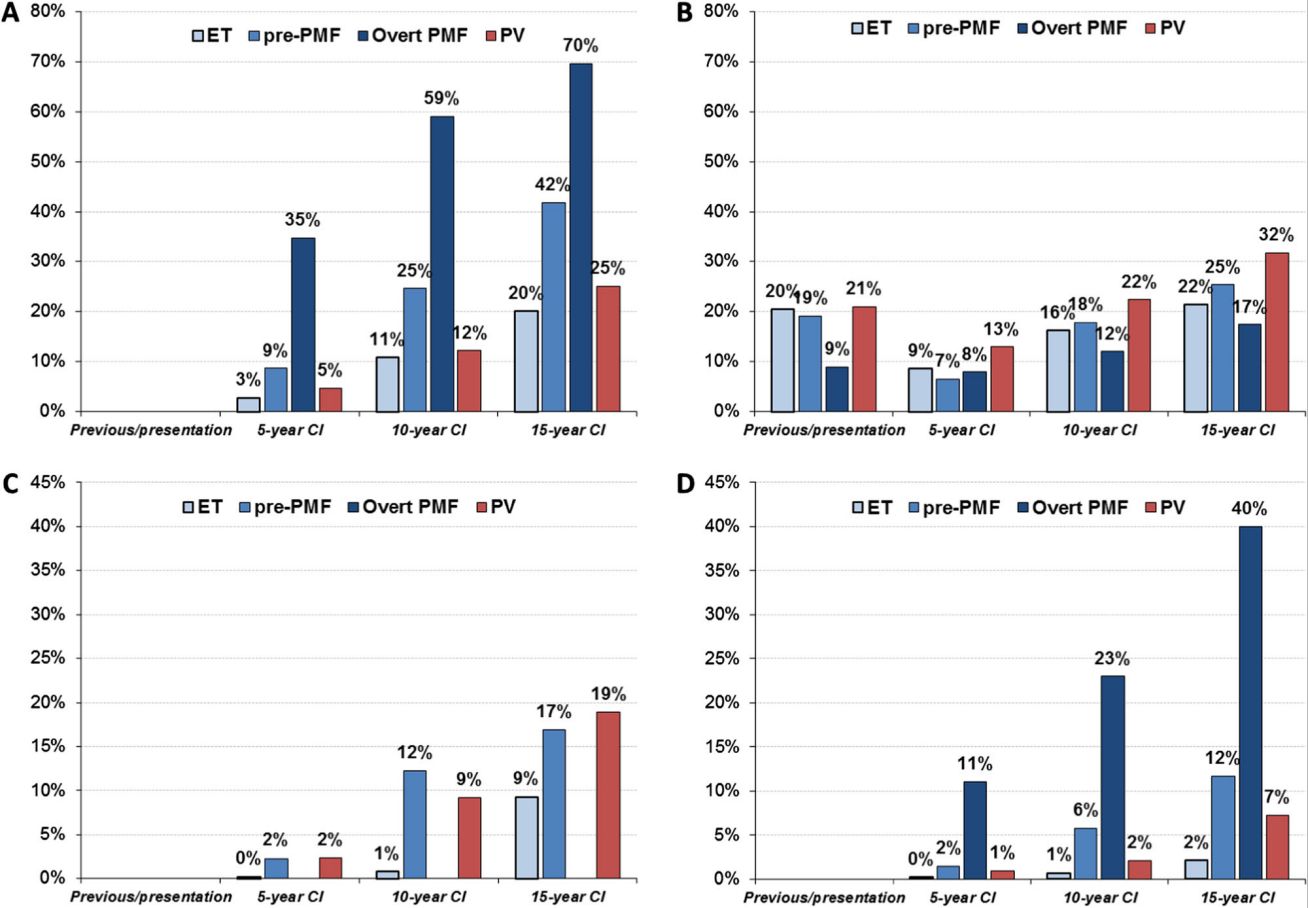
Mortality **a**, major arterial and venous thrombotic complications **b**, myelofibrosis **c**, and Blast transformation **d** in ET, Pre-PMF, overt PMF and PV cohorts. Prevalence of previous events and cumulative incidence (CI) during follow-up calculated at 5, 10, and 15 years from diagnosis. For PMF, two different data sets were considered: *n* = 707 for panel **a**, **b**^18^ and *n* = 383 for panel **d**^14^ and regarding PV for all panels^110^

The combination of clinical, morphological, and molecular genetic features is thought by the WHO as the most suitable attempt to define disease entities such as MPNs (Tables 1 and 2)^1,2, 12^. Following the updated 2008 WHO classification^12^, a number of clinical–pathological studies conducted by different groups have validated these diagnostic guidelines including the importance of morphological features^13–22^. However, a balanced and evidence-based discussion concerning these diagnostic criteria persists^23^. In particular, it has been postulated that ET, PV, and PMF cannot be strictly discriminated by BM morphology as postulated by the WHO, owing to their mimicry to transform to each other^24, 25^. It was argued that *JAK2*-mutated ET resembles PV for similarities of hematological presentation and incidence of clinical manifestations. It is important that this notion should be revisited as the results refer to patients diagnosed with not strictly based WHO criteria^2, 12^. As an example, in a cohort of 466 *JAK2*-mutated ET patients a cumulative risk of evolution to PV from ET was found in 29% at 15 years^25^. However, when strictly adhering to the WHO criteria, the rate of transformation of ET into PV after two decades of follow-up, was rarely documented and accounted for a rate of 1% and only up to 5% of wild type and *JAK2*-mutated ET, respectively^26–29^. The diagnostic differentiation between ET and pre-PMF is not only supported by characteristic morphological BM features of the two diseases but it is also highlighted by the different clinical behavior as reported in Fig. 1a–d. ET is the more benign entity in terms of survival, progression to myelofibrosis (MF) and transformation to blastic phase. Instead the cumulative incidence of major thrombosis in ET is comparable to pre-PMF and lower than PV. On the other hand, pre-PMF has a clear distinct clinical pattern of evolution from ET in terms of evolution into overt PMF, blast crisis, and mortality (Fig. 1a, c, d) and, as previously reported, increased bleeding tendency^22^. In PV, that in the current classification^1, 2^ also includes cases with a prodromal/masked phase (mPV)^30^ there is a trend, in comparison with the other entities, to more frequent thrombotic events and of higher incidence of progression to MF. In overt PMF rates for mortality and transformation to blast crisis are the highest of all MPN subtypes under study, whereas the cumulative incidence of thrombotic complications is lower (Fig. 1a, b, d).

**Table 1:** 2016 World Health Organization diagnostic criteria for polycythemia vera and essential thrombocythemia

	***Polycythemia vera (PV)*** ^a^	***Essential thrombocythemia (ET)*** ^b^
*Major criteria*		
1	Hemoglobin > 16.5 g/dL(men) Hemoglobin > 16.0 g/dL (women) or Hematocrit > 49% (men) Hematocrit > 48% (women) or increased red cell mass (RCM)^c^	Platelet count ≥ 450 × 10^9^/L
2	BM biopsy showing hypercellularity for age with trilineage growth (panmyelosis) including prominent erythroid, granulocytic and megakaryocytic proliferation with pleomorphic, mature megakaryocytes (differences in size)	BM biopsy showing proliferation mainly of the megakaryocyte lineage with increased numbers of enlarged, mature megakaryocytes with hyperlobulated nuclei. No significant left-shift of neutrophil granulopoiesis or erythropoiesis and very rarely minor (grade 1) increase in reticulin fibers^d^
3	Presence of *JAK2* or *JAK2* exon 12 mutation	Not meeting WHO criteria for *BCR-ABL1 + *CML, PV, PMF, MDS, or other myeloid neoplasms
4		Presence of *JAK2*, *CALR* or *MPL* mutation
*Minor criteria*		
1	Subnormal serum erythropoietin level	Presence of a clonal marker (e.g., abnormal karyotype) or absence of evidence for reactive thrombocytosis

Table adapted from Barbui T et al. *Blood Cancer J* 2015; 5:e337^103^ and Arber et al. *Blood* 2016;127:2391–2405^2^

*BM*, bone marrow; *CML*, chronic myeloid leukemia;* MDS*, myelodysplastic syndrome

^a^PV diagnosis requires meeting either all three major criteria or the first two major criteria and one minor criterion

^b^ET diagnosis requires meeting all four major criteria or first three major criteria and one minor criterion

^c^More than 25% above mean normal predicted value

^d^Grading of BM fibers^87^

Criterion number 2 (BM biopsy) may not be required in cases with sustained absolute erythrocytosis: hemoglobin levels. 18.5 g/dL in men (hematocrit, 55.5%) or 16.5 g/dL in women (hematocrit, 49.5%) if major criterion 3 and the minor criterion are present. However, initial myelofibrosis (present in up to 20% of patients) can only be detected by performing a BM biopsy; this finding may predict a more rapid progression to overt myelofibrosis (post-PV MF)

**Table 2:** 2016 World Health Organization diagnostic criteria for primary myelofibrosis

	***Primary myelofibrosis (PMF)*** ^a^	
	**Prefibrotic/early PMF (pre-PMF)**	**Overt PMF**
*Major criteria*		
1	Megakaryocytic proliferation and atypia^b^, without reticulin fibrosis > grade 1^c^, accompanied by increased age-adjusted BM cellularity, granulocytic proliferation and often decreased erythropoiesis	Megakaryocyte proliferation and atypia^b^ accompanied by either reticulin and/or collagen fibrosis (grade 2 or 3)
2	Not meeting WHO criteria for *BCR-ABL1 + *CML, PV, ET, MDS, or other myeloid neoplasm	Not meeting WHO criteria for *BCR-ABL1 + *CML, PV, ET, MDS or other myeloid neoplasm
3	Presence of *JAK2, CALR, or MPL* mutation or in the absence of these mutations, presence of another clonal marker^d^ or absence of minor reactive BM reticulin fibrosis^e^	Presence of *JAK2*, *CALR*, or *MPL* mutation or in the absence, the presence of another clonal marker^d^ or absence of evidence for reactive BM fibrosis^f^
*Minor criteria*		
1	Presence of one or more of the following, confirmed in two consecutive determinations:	Presence of one or more of the following confirmed in two consecutive determinations:
	• Anemia not attributed to a comorbid condition	• Anemia not attributed to a comorbid condition
	• Leukocytosis ≥ 11 × 10^9^/L	• Leukocytosis ≥ 11 × 10^9^/L
	• Palpable splenomegaly	• Palpable splenomegaly
	• LDH level above the upper limit of the institutional reference range	• LDH level above the upper limit of the institutional reference range
		• Leukoerythroblastosis

Table adapted from Barbui T et al. *Blood Cancer J.* 2015; 5:e337^103^. and Arber et al. Blood 2016;127:2391–2405^2^

*BM*, bone marrow; *CML*, chronic myeloid leukemia; *MDS*, myelodysplastic syndrome; *LDH*, serum lactate dehydrogenase

^a^Diagnosis of prefibrotic/early PMF requires all three major criteria and at least one minor criterion. Diagnosis of overt PMF requires meeting all three major criteria and at least one minor criterion

^b^Small-to-large megakaryocytes with aberrant nuclear/cytoplasmic ratio and hyperchromatic and irregularly folded nuclei and dense clustering

^c^In cases with grade 1 reticulin fibrosis^87^, the megakaryocyte changes must be accompanied by increased BM cellularity, granulocytic proliferation, and often decreased erythropoiesis (that is, pre-PMF)

^d^In the absence of any of the three major clonal mutations, the search for the most frequent accompanying mutations *(ASXL1, EZH2, TET2, IDH1/IDH2, SRSF2, SF3B1*) are of help in determining the clonal nature of the disease

^e^Minor (grade 1) reticulin fibrosis secondary to infection, autoimmune disorder or other chronic inflammatory conditions, hairy cell leukemia or other lymphoid neoplasm, metastatic malignancy, or toxic (chronic) myelopathies

^f^BM fibrosis secondary to infection, autoimmune disorder, or other chronic inflammatory conditions, hairy cell leukemia, or other lymphoid neoplasm, metastatic malignancy or toxic (chronic) myelopathies

### Rationale behind the 2016 changes in the diagnostic criteria for PV, ET, PMF, and pre-PMF

In comparison with the 2008 WHO guidelines^12^, several important improvements mostly derived from clinic-pathological and molecular genetic studies have been highlighted:

(i) Discovery of novel molecular findings that provide deeper insights for the understanding of the pathobiology of MPNs that are in keeping with clonality^31^ and exert an impact on diagnosis^26, 32^ and outcome^14,15, 26^.
(ii) Lowering of the diagnostic hemoglobin (Hb)/hematocrit (Hct) threshold values with introduction of mPV that has changed markedly the diagnostic landscape of this MPN subtype and consequently options for treatment and outcome^30, 33–35^ by revealing that PV has been underdiagnosed in the past^34, 36^. In this context, BM histology was promoted from a minor to a major diagnostic criterion by recognizing its reproducible characteristic morphological features^37–40^.
(iii) Emphasizing the need to discriminate “true” ET from pre-PMF by an accurate evaluation of BM biopsy features^41^, including the lack of reticulin fibrosis at onset in <5% of cases, which has been formerly neglected^42^. It can only be underscored that this distinction is of significant prognostic and therapeutic relevance^13, 15–18^.
(iv) Advancements regarding the characterization and standardization of morphological BM features yielded an improvement in the differentiation of MPN subtypes, particularly between ET, pre-PMF, and PV^17,20,43, 44^. The latter presents one of the critical key issues for hemato-pathologists to improve their agreement rates (up to ~ 80% depending on study design)^17–19, 45, 46^ and decrease the number of unclassifiable cases (currently down to maximal 5%)^47^.

Following the 2016 revision^1, 2^ of the 2008 diagnostic guidelines proposed by the WHO^12^, critical questions were still raised and reflected by comments in recently published reviews on MPNs^48, 49^. In one of these reviews^48^ these refer to the presentation of borderline expressed so-called minor clinical criteria in pre-PMF^15^ or the Hb threshold values necessary to diagnose PV^34^. More general arguments are related to the failing diagnostic specificity of BM morphology for differentiation of MPNs, except that myelodysplasia can be ruled out on the basis of histologic features^49^. Erroneously, it is assumed that the transformation of MPN demonstrates that diagnosis is a moving target^49^. According to the WHO classification^1,2, 12^ mPV may initially mimic ET and therefore usually transforms later to overt PV^27,50, 51^ or pre-PMF may present with an ET-like phenotype and may progress to overt PMF^13,15, 18^. In aggregate, these so-called instabilities of subtyping MPNs are significantly dependent on the accuracy of initial diagnosis^27^.

### The complementary role of mutation screening and its limitations for diagnostic purposes

The 2008 WHO classification of MPN was largely inspired by the discovery of mutations in *JAK2* (chr. 9p24), namely V617F^52–55^ in exon 14 and *indels* in exon 12^56^, and *MPL* (chr. 1p34), mainly at codon W515^57^, that were incorporated as major diagnostic criteria^12^. The *JAK2*V617F is the most prevalent mutation in MPN, accounting for ~ 95% of PV and 60% of ET and PMF. Variable deletions and insertions clustering at codon 537–543 in exon 12 of *JAK2* are detected in ~ 3–5% of patients with *JAK2*V617F unmutated PV by using sensitive approaches, as mutation allelic burden in whole blood and purified granulocytes is low^58^. Mutations in *MPL* cluster in exon 10 at codon 515, the most prevalent being a W to K, L, A, R transversion, and rarely at codon 505 (S > N), originally reported in familial cases of thrombocytosis^59^. They are found in ET and PMF with approximate incidence of 4 and 8%^60^. Finally, in 2013, mutations in *CALR* (chr. 19p13.2), the gene encoding the endoplasmic reticulum-associated chaperone calreticulin, were detected in patients with *JAK2/MPL* unmutated ET and PMF^61, 62^. These are highly heterogeneous *indels*, all clustering in exon 9 that encodes for the C-terminus portion of the protein. There are two prevalent (>80% of all *CALR* variants) mutation types, type 1 (a 52-bp deletion; p.L367fs*46) and type 2 (a 5-bp insertion; p.K385fs*47), whereas the remaining are defined as type 1-like and type 2-like based on predicted helix propensity similarities with the former^63^. The type 2 *CALR* mutations are preferentially associated with ET, whereas type 1 predominates in PMF. The above three driver mutations are listed as major criteria for PV (*JAK2*V617F and exon 12), ET, and PMF (*JAK2*V617F, *CALR* and *MPL*) in the revised 2016 classification^1, 51^. Therefore, the modern diagnostic approach to MPN requires the knowledge of mutation status^64^. However, in the instances when genotyping for these mutations is not available, or the mutations result absent in diagnostic samples, minor criteria in the WHO classification are included to support diagnosis otherwise. Some PV patients who lack *JAK2* mutations might eventually harbor other mutations in *JAK2*^64^ or other genes such as *SH2B3/LNK*^65^. On the other hand, up to 20% of ET and 10–15% of PMF patients have no driver mutations, and are currently referred as “triple-negative” (TN); some of these case have non-canonical mutations in *MPL* and *JAK2*, but overall they do not account for >10% of the TN category^66, 67^. For triple-negative PMF patients, the 2016 WHO classification supports the search for other non-driver “most frequent” mutations, e.g., in *ASXL1*, *EZH2*, *TET2*, *IDH1/IDH2*, *SRSF2*, *SF3B1*, that if present stand as a marker of clonality. These mutations lack both disease specificity and mutual exclusivity; however, they are found in ~ 50% of cases with PMF^14, 68^ and using wider amplicon panels up to 81% of the patients presented one clonal marker^69^. Although not explicitly stated in the WHO classification, also chromosomal abnormalities might serve as marker of clonality. Presence of the above additional mutations is not currently included as criteria of clonality in cases of PV or ET lacking driver mutations, although a recent large study showed that ~ 50% of the patients had at least one such mutations^70^. Interpretation of these genetic variants is complicated by the discovery of CHIP, “clonal hematopoiesis of indeterminate potential”, that reflects the “trending toward inevitability”^71^ age-related accumulation of mutations^72, 73^; however, in the context of hematologic abnormalities that characterize MPN patients, finding any of these mutations certainly is in favor of the existence of a pathologic clonal hematopoiesis.

### Current controversies regarding the new diagnostic criteria, especially in regards to diagnosis of PV and prefibrotic PMF

Serious concern has been expressed by several authors regarding the lowering of the diagnostic Hb threshold values (>16.5 g/dL for men and >16.0 g/dL for women) proposed by the 2016 revision by the WHO^1, 2^ for the diagnosis of PV^36,74, 75^. The main points of criticism are that these new criteria will lead to unnecessary and costly investigations including a large segment of the healthy population^36, 74^. To evaluate the proportion of presumptive PV by strict application of the 2016 WHO criteria regarding the low Hb thresholds^1, 2^, a retrospective analysis of the complete blood cell count (CBC) was performed on very large cohorts of unselected subjects^36, 74^. Following this scotom-like focus on one single parameter gained from routinely done CBCs in the Canadian population ~ 4.1% of the males and 0.35% of the females revealed these Hb threshold values^36^ compared with the Brazilian population with ~ 5.6% males and 0.22% females^75^. These data would imply that the annual incidence of potential PV patients may increase by up to 12-fold in males and threefold in females^36, 74^. However, it has to be noted that these data were derived from routinely performed CBCs, without any knowledge about the JAK2 mutation status and were not obtained from clinic–pathological databases as the WHO threshold values for Hb^74^.

In contrast, patients presenting with mPV^30, 33^ showed that many cases as defined by the WHO 2016 criteria^1, 2^ were actually missed. A study on 118 patients with mPV included 72% cases with a history of previous arterial and /or venous thrombosis and according to the applied CBC parameters showed thrombocytosis as being the most frequent finding with 64% (either isolated or combined with leukocytosis)^34^.

Thrombocytosis presents an important issue concerning the differentiation between mPV and ET^50, 51^ that has been already recognized before the establishment of the 2016 WHO revision^1, 2^ and was further emphasized regarding therapeutic consequences^76^. Misdiagnosis of mPV for ET implies that phlebotomies will erroneously not be considered^36^. In this context it should be underscored that PV patients require phlebotomies to a therapeutic Hct target of <45%^77, 78^. Summarized, recognition of early stages of PV is in keeping with a major advancement in the field of MPNs and will certainly avoid underdiagnosis by preventing fatal thrombotic events and initiation of proper treatment^30,35,36, 78^.

Current problems associated with pre- PMF and ET start with the fact that existence of a pre-PMF is not everywhere recognized, although as the first descriptions in the late nineties^13^ its existence including its clear differentiation from ET^79, 80^ has been demonstrated. Following a lively discussion in the past years^38^ pre-PMF was definitely confirmed by several groups^14,15,38, 81–85^ but until now not regarded by the updated British guidelines^16, 86^. According to the 2008/2016 WHO classification^1,2, 12^ pre-PMF may present either with no increase (fiber grade 0) or minor grade of reticulin fibrosis (fiber grade 1)^87^, whereas overt (classical) PMF is characterized by fiber grades 2 and 3 including collagen^88^. Difficulties to accept pre-PMF as clinically relevant entity may be caused by the fact that diagnosis of pre-PMF was predominantly based on morphological characteristics and that presenting clinical features may be different depending whether pre-PMF patients were collected from cohorts with an ET-like phenotype^15,17, 18^ or with features resembling a more PMF-like phenotype^85^ without thrombocytosis^14^. Molecular markers of pre-PMF are different from ET, but their discriminant power is relatively low^13, 26^.

Recent investigations confirm that clinical presentation of pre-PMF is different from ET and this may influence therapeutic decision making and outcome. Ample evidence has been provided by several groups that an accurate discrimination between pre-PMF and ET is not trivial^13^ but has an impact not only on presenting laboratory data but also on complications like disposition to hemorrhage, thrombosis, and outcome with progression to overt myelofibrosis, transformation to blast crisis, and overall survival^15–18, 32^.

Laboratory data at initial diagnosis are of distinctive impact between pre-PMF and WHO-defined ET as it is shown that at least one of the minor criteria for diagnosis of pre-PMF defined by the WHO (anemia, leukocytosis, elevated LDH levels, and splenomegaly) is highly prevalent with 91% in pre-PMF in comparison with 48% in ET^15^. Greater values of circulating CD34 cell count in pre-PMF in comparison with ET and a significantly more active *in vitro* stem growth in peripheral blood MNCs from pre-PMF are valid parameters for a different biological behavior^15,17,18,38,83, 89^. This is in line with the observation of a prognostic unfavorable impact of the *JAK2*V617F mutation in pre-PMF versus a more benign course of disease in patients with a CALR mutation, which could not be seen in ET patients strictly diagnosed by WHO criteria^15^.

To investigate if blood tests can exert a predictive power in patients presenting clinically with an ET-like phenotype Hb value, WBC count and LDH level were used in a dichotomized fashion, resulting in a step-by-step procedure. Utilizing this algorithm provided a sensitivity and specificity of ~ 50%^90^. To confirm and improve this investigation by expanding the so-called Bergamo algorithm regarding its discriminatory ability, a novel logistic regression model was introduced generating a substantial increase in sensitivity and specificity to ~ 75%^91^. In aggregate the authors of this investigation concur that although BM biopsy examination persists to remain an integral part of the final diagnosis, laboratory parameters at presentation may provide clinicians with additional information to suspect pre-PMF in a patient with a presumptive clinical diagnosis of ET.

Regarding the rates for survival, blast transformation (acute leukemia), and progression to overt myelofibrosis data were significantly worse in pre-PMF compared to WHO-confirmed ET^13–15, 17,18,38, 92–94^. Moreover, the striking differences in clinical phenotypes between pre-PMF and overt PMF may not allow to use the risk scoring systems established for overt PMF for decision making in pre-PMF^84^.

Finally, the different clinical picture and outcomes in pre-PMF and ET result in different treatment needs. This is impressively demonstrated by different treatment outcomes when hydroxyurea was prospectively compared with anagrelide in ET patients diagnosed according to the PVSG criteria (designating many pre-PMF patients as ET) with an advantage for hydroxyurea in the UK-PT1 study versus the same comparison in WHO-classified ET in the anahydret study with an equal efficacy of anagrelide^21, 95^.

### Ongoing controversies with regard to BM morphology in the diagnosis of MPN subtypes

It has been argued that performing a BM trephine biopsy in *JAK2*-mutated patients with sustained absolute erythrocytosis with Hb concentrations of >18.5 g/dL in men or >16.5 g/dL in women or Hct >55.5% in men or >49.5% in women, might be associated with some hazards for the patient and is not warranted. In addition, it has been argued that morphology in general does not provide enough diagnostic specificity for the differentiation of PV from other types of MPN, nor does provide useful prognostic information^49^. The concerns in relation to complications related to BM biopsy seems to be unsubstantiated^96^.

A recent blinded review study has shown that characteristic BM features of PV are highly reproducible with an overall interobserver agreement of almost 93%^39^. Interestingly, this series did include specimens of mPV, overt PV, and *JAK2*-mutated ET, as well as other *JAK2*-mutated patients that did not meet the 2008 WHO threshold^12^ for an elevated Hb level but were confirmed as PV based on their increased red cell mass^40^. BM biopsy is also capable of providing prognostic information. This is particularly true for the identification of BM fibrosis^87, 88^. Although a variable incidence and severity of BM fibrosis has been reported in the past, it has to be emphasized that most of these older studies included advanced disease stages more consistent with post-PV myelofibrosis^97^ presenting with grades 2 and 3 of reticulin/collagen fibrosis^98, 99^. The clinical impact and prognostic relevance of the presence at disease outset of reticulin fibrosis^38^ has been demonstrated in > 500 patients with WHO-defined PV who were strictly evaluated at time of initial diagnosis. In this study, grade 1 reticulin fibrosis^87^ was found in 14% of patients and in only two cases a higher grade could be observed^100^. In general, clinical and laboratory characteristics did not differ between patients with or without BM fibrosis, however, a significant higher prevalence of palpable splenomegaly was observed in cases with BM fibrosis, and most importantly, patients presenting with initial fibrosis transformed more frequently into post-PV myelofibrosis^100^. These data were recently validated by emphasizing the association between BM reticulin fibrosis at onset of PV and subsequent fibrotic progression^101^. In addition, palpable splenomegaly and leukocytosis were also identified as important risk factors^101^. For this reason, evaluation of a BM biopsy specimen in PV validates not only the accurate diagnosis, especially in doubtful cases^78^, but also provides important information concerning progression to post-PV myelofibrosis (spent phase). Altogether, the recognition that PV is characterized by a specific histological BM pattern^37–40, 92, 102^, allowed the “promotion” of BM histology to one of the major diagnostic criteria in the 2016 WHO revision^1, 2^. Accordingly, BM biopsy examination was recommended to be performed in a recently published practical diagnostic algorithm for PV and secondary polycythemia^78^.

Discussion and controversies persists that histological criteria characterizing the specific MPN subtypes of pre-PMF and ET, as described by the WHO classification^1,2,12, 103^ are difficult to apply, and thus unreliably reproducible in routine practice. It has been postulated that a more objective, algorithmic-based procedural approach that also include a quantitative assessment of individual morphological features^13, 41^ should instead be applied to achieve a clearer separation of true ET from pre-PMF. It should be noticed, however, that the diagnosis of specific subtypes, in particular in early stages, is not captured by single morphological parameters^92^, but must takes into account the entirety of the complex BM architecture in MPN, which is best captured by specific diagnostic patterns^21, 43^. In relation to PMF, it is important to realize that grading of BM fibrosis has a significant impact on clinical presentation and overall outcome^14,104, 105^. Moreover, regarding ET a major advancement of the 2016 WHO revision^1, 2^ was to clarify incidence and the maximum grade of reticulin fibrosis seen in this disease at its outset to strengthen the differentiation from PMF^15,18, 38^. Reproducibility of WHO-defined morphological features for the differentiation of ET from pre-PMF has been evaluated by studying large cohorts of patients with varying numbers of involved panelists with or without prior knowledge of clinical data. In aggregate, > 80% (range 76–88%) diagnostic consensus with formal assessment of interobserver variability was reached in 2033 patients derived from several independent study groups^17–19, 45^. It has to be stressed that for the first time in one of these studies, specimens representing a wide spectrum of reactive lesions as well as normal BM and all major subtypes of MPNs were included to more closely reflect a “real world” pathology setting, i.e., daily routine^19^. Referring to the reliability to reproduce the postulated WHO guidelines^1,2, 12^ the group of unclassifiable MPNs (MPN-U) has to be briefly discussed herewith. The proportion of cases that a given pathology deem to be “unclear” and thus allocates to the MPN-U group, may be considered as a true yardsticks for the accuracy to discriminate MPN subtypes. Reported incidence of MPN-U varies significantly in different studies with a range up to > 20%^19, 106^. However, most studies show an incidence of 10–15% or even less^19,38, 107^. When the 2016 WHO criteria^1, 2^ have been applied the incidence is reduced to <5%^47^. These conspicuous differences may be significantly related to the differences in experience of the reviewer, a high incidence of cases of MPN presenting in very early phase, preceding cytoreductive treatment which may have affected the morphologic findings and/or incomplete clinical data and mutation status knowledge^92^.

### Challenges in applying the WHO criteria in routine clinical practice, and possible future directions

The 2016 revised WHO classification^1, 2^ is supposed to have immediate routine application, in particular regarding the early diagnosis of PV and a clearer-cut distinction between pre-PMF and both ET and overt PMF, as such distinction has important outcome correlates^14,15, 85^. The value to recognize early and distinct phases of diseases, through the characterization of as homogeneous as possible clinical, histopathology and molecular patterns (for example, *CALR* mutation is very unlikely to indicate PV, if not exceptionally^108^, and *MPL* mutation virtually negates it), is projected to improve the management and hopefully the outcome.

With this in mind, we think that the adoption of the revised 2016 WHO criteria^1, 2^ in the clinical practice as a “state-of-the-art” approach, yet in an ever changing research scenario, will be the best way to collect homogenously defined categories of patients for assessing their clinical course, outcome, response to conventional and new target therapies and, not by least, provide material for further molecular and cellular studies aimed at discovering surrogate diagnostic biomarkers. Gene and/or non-coding small RNA expression profiles in the context of selected mutation patterns, Nano-String interrogation of BM tissues, levels and types of inflammatory cytokines and chemokines, cell membrane antigen combinations, are all fields of investigation that have the chance to delineate integrated patterns. These novel techniques may eventually replace BM biopsies that, however, presently stand as a stone regarding the modern diagnostic approach to MPN.

### Challenges in applying the WHO criteria in routine clinical practice–future directions from the perspective of the hematopatholgist

The reproducibility of the histological characteristics as described in the WHO classification remains a debate issue^49^. Although the overall histological evaluation shows a high degree of reproducibility, the identification of specific morphological features displays a more limited reproducibility among different hemato-pathologists. The level of consensus has revealed a wide range between 49 and 100% in some studies^45^. Several reasons acting alone or in concert may be hypothesized to account for these shortcomings: (1) failure to reproducibly identify standard BM features of distinctive diagnostic value^43, 44^ undermining a correct morphological interpretation;^17,19,39, 92^ (2) inclusion of small, non-representative biopsy specimens with extensive crushing artefacts or fragmentation; (3) disregard of age-related adjustment for assessing hematopoietic cellularity^87^; (4) inability of performing an accurate fiber grading owing to a variety of staining artefacts^86, 106^; (5) unexperienced investigators^45^.

A central pathology review may be desirable in some clinical settings. Although BM fibrosis grading is considered a part of a standard BM biopsy examination report, low overall level of concordance of only 55.8% (range 33–100%) on 579 biopsy specimens between the local pathologists from various countries and a central review evaluation was demonstrated by a recent study^109^. This is in sharp contrast with central pathology review rates of 83–99.7%; these rates of > 80% are considered to represent excellent agreement according to the standards used to measure the strength of concordance^46^.

All those issues are resolvable. In this regard, it has been demonstrated that the weighting of individual features defining a morphological pattern can be substantially affected by training sessions^45^. Educational seminars and workshops for hemato-pathologists can significantly improve the integration of all histological characteristics into a meaningful, reproducible subtyping of MPNs^20, 45^. This includes an increased consensus on the identification of pre-PMF^84^.

In addition to distinguishing between the different subtypes of MPNs, their separation from MDS/MPN overlap syndromes or MDS particularly in clonally undefined (triple-negative) PMF has proven to be of upmost clinical importance as the outcome for the different subtypes varies significantly^14,47, 92^.

## Conclusions

The WHO committee of hemato-pathologists, clinicians, and scientists with special interest in MPN has now delivered the most comprehensive and practically useful outline of diagnostic criteria for ET, PV, and PMF. The authors of the current review strongly recommend the collection of BM examination at time of diagnosis of MPN and encourage repeating the procedure during follow-up, in the presence of signs of progressive disease. In all instances, sufficient BM aspiration should be secured in order to allow screening for driver and other mutations, as well as cytogenetic analysis.

Special attention to morphology is required in order to distinguish ET from pre-PMF and *JAK2*-mutated ET from PV. Such details are prognostically relevant as survival has been shown to be the longest in strictly WHO-defined ET, whereas it was significantly worse in pre-PMF and PV. BM examination is also the most optimal method of obtaining cytogenetic information that has been shown to influence survival in both PMF and PV. Establishing driver mutational status in patients with MPN is not only important in complementing morphologic diagnosis but also provides important prognostic information.

In regards to diagnosis, PV is expected to be almost always accompanied by a *JAK2* mutation, whereas the specific driver mutation cannot otherwise distinguish one MPN from another; however, in distinguishing ET from pre-PMF or mPV, a higher *JAK2*V617F allele burden favors the diagnoses of the latter rather than the former. In terms of prognosis, thrombosis risk in ET is strongly tied to the presence of *JAK2* mutations, whereas the presence of type 1/like *CALR* mutations in PMF portends superior survival. In the future, we expect an increasing role for other mutations in complementing morphologic diagnosis in MPN and providing additional prognostic information.
